# From society to cell: Exploring the biological impacts of social exposures through linked biological and population-level child development data.

**DOI:** 10.23889/ijpds.v7i3.2074

**Published:** 2022-08-25

**Authors:** Kimberly Thomson, Monique Gagne Petteni, Anne Gadermann

**Affiliations:** 1 University of British Columbia

## Objectives

Our objective was to capture a holistic view of a child from “society to cell” by building an inter-disciplinary data linkage between social, biological, and environmental factors to better understand how “experience gets under the skin” to influence social disparities in child development from conception onwards.

## Approach

Linking a unique combination of administrative, survey, and biological data, this research connects children’s epigenetic profiles and other biological markers (e.g., microbiome) to broader social determinants of health to examine the process of “biological embedding.” In British Columbia, Canada, the CHILD Study has collected biological data for a cohort of children from birth to age 5 (N = 840). Linked administrative records (e.g., health services, demographic data) provide key social and environmental information (e.g., parental depression, neighbourhood socio-economic status). Population-level child development data have been collected at age 5 using the Early Development Instrument and are linkable for a subset of this cohort (N ~ 250).

## Results

This unique linkage helps us understand the critical interplay between health outcomes and resulting disparities; how a child’s social environment may impact their biological makeup, and in turn their health and developmental outcomes due to influences on their neural, endocrine, and/or immune systems at the molecular level. Rather than limiting research to singular disciplines, this research aims to shift thinking around health problems towards a synthesized model to examine mechanisms and associations between early experiences and biological outcomes. We will discuss strengths and challenges of developing this linkage and working across disciplines such as medicine, education, and science to address research questions that span historically disparate areas of research.

## Conclusion

An interdisciplinary approach has been essential for the development and approvals of this project. Bringing together experts from diverse disciplines with different perspectives to use a novel approach enables us to better address the large and important issue of childhood social disparities and their enduring impact on life course health.

